# Processing Speaker-Specific Information in Two Stages During the Interpretation of Referential Precedents

**DOI:** 10.3389/fpsyg.2020.552368

**Published:** 2020-11-23

**Authors:** Edmundo Kronmüller, Ernesto Guerra

**Affiliations:** ^1^Escuela de Psicología, Pontificia Universidad Católica de Chile, Santiago, Chile; ^2^Centro de Investigación Avanzada en Educación, Instituto de Educación, Universidad de Chile, Santiago, Chile

**Keywords:** language processing, reference, speaker specificity, perspective taking, pragmatics, eye tracking

## Abstract

To reduce ambiguity across a conversation, interlocutors reach temporary conventions or *referential precedents* on how to refer to an entity. Despite their central role in communication, the cognitive underpinnings of the interpretation of precedents remain unclear, specifically the role and mechanisms by which information related to the speaker is integrated. We contrast predictions of one-stage, original two-stage, and extended two-stage models for the processing of speaker information and provide evidence favoring the latter: we show that both stages are sensitive to speaker-specific information. Using an experimental paradigm based on visual-world eye tracking in the context of a referential communication task, we look at the moment-by-moment interpretation of precedents and focus on the temporal profile of the influence of the speaker and linguistic information when facing ambiguity. We find two clearly identifiable moments where speaker-specific information has its effects on reference resolution. We conclude that these two stages reflect two distinct cognitive mechanisms, with different timings, and rely on different representational formats for encoding and accessing information about the speaker: a cue-driven memory retrieval process that mediates language processing and an inferential mechanism based on perspective-taking abilities.

## Introduction

Definite repeated reference is a ubiquitous phenomenon during a conversation. Interlocutors talk about uniquely identifiable entities that are referred to multiple times using the same or a similar expression, such as “the black guitar,” “my classmate,” or “your boss.” Because each entity can be referred to in multiple ways (e.g., “the old guitar,” “she,” or “Mrs. Smith”), and the same expression can be used to refer to different things (there are many black guitars, as well as classmates and people whose last name is Smith), interlocutors reach temporary conventions on how to refer to each entity. These conventions or referential precedents help in reducing ambiguity (Clark and Wilkes-Gibbs, 1986; Brennan and Clark, 1996). Therefore, speakers tend to use the same expression, and listeners expect that expression to be used to refer to the same entity across a conversation. Despite the central role that this phenomenon plays in communication and the considerable amount of research done to explain it, the cognitive underpinnings of the interpretation of precedents remain a matter of debate, specifically concerning the mechanisms by which information related to the speaker is integrated.

One proposal is that interpreting precedents is a *one-stage* process resulting from the functioning of a single language processing mechanism that integrates speaker, linguistic, and all other available contextual information, as soon as it is available, and with little or no delay in this availability (Metzing and Brennan, 2003; Brown-Schmidt, 2009). This one-stage process is in line with constraint-based models postulated in the context of sentence processing and definite reference resolution (McRae et al., 1998; Spivey and Tanenhaus, 1998; Hanna et al., 2003).

An alternative proposal postulates two stages. The first one is accomplished by an initial interpretation relying solely on linguistic information, and the second one is achieved by a perspective-taking mechanism, which relies on speaker-specific information, and it is triggered on demand for correcting potential misunderstandings (Barr and Keysar, 2002; Kronmüller and Barr, 2007). This *original two-stage* approach is in line with an inference making mechanism that “anchors” an initial interpretation egocentrically and later “adjusts” based on mutual knowledge and speakers’ beliefs (Keysar et al., 1998; Keysar et al., 2000; Epley et al., 2004). The following sections present a discussion of the existing literature that has been previously taken as support for either of these accounts of the online interpretations of precedents and offers an alternative to both proposals: an *extended two-stage* account.

### Referential Precedents and Speaker Specificity

What a speaker wants to achieve when uttering a referring expression is to bring a specific entity into joint attention. The listener, in turn, should go beyond the conventional meanings of the words in the referring expression and consider background information, such as the time and place of the interaction, the goals of the conversation, and, critically, the identity and shared history with the speaker (Strawson, 1950).

An initial account on how reference resolution is achieved, under both the time constraints of a conversation and the cognitive demands on the language processing system, postulates an intrinsic context for comprehension. This context does not comprise all possible background information but only information shared among interlocutors and known as shared (Clark and Marshall, 1981; Clark and Carlson, 1981). It is by this mutual knowledge or *common ground* that a definite reference can point toward a unique referent; it narrows down the possible alternatives that otherwise would be many. Indeed, the same expression (e.g., “the black guitar”) can be used to refer to many different objects in the world, but only to one—or a few—when the referential domain is restricted to the guitars that are mutually known by the interlocutors.

The common ground view is applicable to explain how referential precedents work; in fact, referential precedents can be considered a paradigmatic case for testing it. Under this view, precedents reflect a *conceptual pact* that is common ground among interlocutors (Brennan and Clark, 1996). Following our example, we can conceptualize a guitar as “black” (instead of “electric,” “old,” or “mine”) and create the precedent “black guitar,” such that it reflects that shared conceptualization. The benefit of using precedents on comprehension, in the strong version of this view, should be specific to the partner of the conversation with whom the pact was reached. To test this prediction, Barr and Keysar (2002) conducted an eye-tracking experiment. They showed that the benefit of precedent use—specifically on the speed of resolving reference as measured by the latency to look at the target object among all other possible objects in the referential domain—was not dependent on the precedent being common ground but in the use of the precedent *per se*.

In the experiment, a listener participant played the role of matcher in a referential communication game. The task was to arrange a set of objects in a 4 × 4 cubbyhole following the instructions from two confederate speakers playing the role of directors. One of the speakers interacted live with the participant, while the other had recorded the instructions previously that were played back through headphones only to the participant. This manipulation generates a situation in which the participant had privileged information regarding the names given to the different objects in the referential domain; in other words, one precedent—the one that was provided by the live speaker—was common ground between the speaker and the listener, but the other was privileged knowledge of the listener. They found that looks to the target object were faster when there was a referential precedent previously established. However, this benefit was independent of whether the precedent was common ground with the speaker or privileged for the listener. Based on this result, they postulated that the processing of referential precedents was *speaker-independent* instead of *speaker-specific*, in the sense that precedents provide a “linguistic index to the representation of the referent in memory” (Barr and Keysar, 2002, p. 392). In their proposal, speaker specificity only appears as the result of a slower adjustment mechanism, specifically perspective taking, that keeps track of common ground and has a role in correcting for potential misunderstandings.

In an influential study, Metzing and Brennan (2003) challenged this conclusion. They postulated that the interpretation of a precedent is guided by memory representations encoding not only the link between a precedent and its referent (as the idea of “linguistic index” suggests) but also a link to contextual features. Among these features is speaker-specific information (e.g., her identity and shared knowledge with the listener). Besides the situations where a precedent was maintained by speakers, as in Barr and Keysar (2002), they added two cases where a precedent was broken either by the original speaker who had established it or a new speaker uninformed of its existence. In one condition, one speaker established a precedent for a strange object (without a conventional name in English) calling it, in a first instance, “the silver pipe,” but in a second instance, “the shiny cylinder.” In the contrasting condition, one speaker called the strange object in a first instance “the silver pipe,” while a second speaker, not informed about the precedent because she was not present at the moment it was established, call it “the shiny cylinder.”

In the first case—when the original speaker breaks the precedent—the authors reasoned that interference, which is manifested in a delayed resolution of reference, should be observed because there are no good reasons for the original speaker to change the previously established precedent. In the second case, when a new speaker breaks a precedent, no interference should be expected because this new speaker could call that object in many different ways, inasmuch as she did not know about the existence of the precedent. They replicated Barr and Keysar’s (2002) results for the maintained case (i.e., the speaker-independent effect of precedent), but they did find speaker specificity when the precedent was broken: listeners took longer to resolve reference when a new expression was used by the original speaker than when the precedent was broken by the new speaker. In terms of cognitive processing, they interpreted their results as reflecting the functioning of a single cognitive mechanism that integrates many different sources of information, which might have different levels of influence on comprehension based on their relative strength. In the case when a precedent is maintained, the effect of the speaker’s identity was overwhelmed by the strength of the linguistic cue, not allowing the former to express. In contrast, when the linguistic cue is not as strong as the speaker identity cue—as in the case when a precedent is broken by presenting a brand new expression—the latter can be expressed, and its influence can be seen on comprehension.

These two studies set the current debate on the cognitive underpinnings of referential interpretation of precedents. It is either a one-stage process, as proposed by Metzing and Brennan, or a two-stage process, as proposed by Barr and Keysar (for an overview of this debate, see Brennan et al., 2010, 2018). In this debate, the main source of disagreement has been whether speaker-specific information influence comprehension at the same moment as linguistic information (Brown-Schmidt, 2009), or whether it plays a secondary role, expressed in a delayed influence compared with the linguistic input (Kronmüller and Barr, 2007). Because timing is at stake, the eye-tracking technique has been crucial since it allows observing the interpretation processes as it unfolds, making possible to determine the moment at which the different sources of information have their influence (Allopenna et al., 1998; Altmann and Kamide, 1999; for a review see Knoeferle and Guerra, 2016).

### The Interpretation of Referential Precedents: One-Stage or Two-Stage?

The one-stage alternative postulates that the language processing system integrates linguistic and speaker information, along with other contextually relevant information, in an online fashion, that is, as soon as the information is available. Thus, the linguistic information carried by a precedent, the information about the speaker, the common ground between interlocutors, and other relevant contextual information all have an immediate effect on the interpretation of that precedent. Evidence for this immediate effect of speaker information has been found in cases where a precedent is maintained: there are faster looks to the intended referent when it is the original speaker that maintains a precedent than when a new speaker does (Barr, 2008; Brown-Schmidt, 2009; Horton and Slaten, 2012). Further evidence for single-stage processing of linguistic and speaker information has been found for definite reference (Hanna et al., 2003) and contrastive definite reference (Heller et al., 2008). There is also convergent electrophysiological evidence showing an early integration of speaker and linguistic information (see Van Berkum et al., 2008) and common neural pathways for processing lexical and speaker characteristics (Tesink et al., 2008).

The original two-stage alternative, in contrast, postulates a first stage where an utterance is processed independently of speaker information (for example, based solely on the linguistic input), followed by a second stage where speaker-specific information is integrated, particularly speaker’s knowledge and beliefs (Kronmüller and Barr, 2007). Under this view, the influence of speaker-specific information should be clearly identifiable but only after the influence of the linguistic input (Barr and Keysar, 2002; Kronmüller and Barr, 2007; Kronmüller et al., 2017). In other words, the effect of who said what should not be seen before the effect of what is said. Evidence for two stages has been found for the cases when a precedent is broken: listeners avoid mapping a new linguistic expression onto a referent that has been named previously, independently of whether or not the previous referent-expression mapping is common ground (Kronmüller and Barr, 2007). Specifically, listeners look away from an object associated with a precedent, independently of whether that precedent was established by the original speaker or the new one (not aware of the existence of the precedent). Only after this initial interpretation will listeners allow a new referent-expression mapping for the new speaker for whom the original mapping was unknown, which is expressed in faster looks to the target with the new speaker than with the original speaker. This late process was characterized as a delayed *recovery* based on common ground information, and importantly, it was completely impaired by cognitive load, suggesting that it is a different cognitive process than the initial interpretation, which is only mildly affected (Kronmüller and Barr, 2007). Online evidence for two distinct stages has also been found for definite reference (Wu et al., 2013) and negated referential expressions (Kronmüller et al., 2017). Finally, brain imaging research has found convergent evidence for two distinct mechanisms for processing linguistic information and the speaker’s beliefs and knowledge (Willems et al., 2010; Bögels et al., 2015).

Considering the wide disagreement between these two alternatives, which is mainly expressed empirically on the moment when speaker-specific information influences the interpretation of precedents (operationalized, as we have described above, as looks to the intended referent or target over time), Kronmüller and Barr (2015) conducted a meta-analysis. In this meta-analysis, they included all the experiments implementing the situations described above: (a) an original (same) speaker maintaining a precedent; (b) a different speaker maintaining a precedent from another speaker; (c) an original speaker breaking his/her own precedent, and (d) a different speaker breaking a precedent established by the original speaker.

**FIGURE 1:**
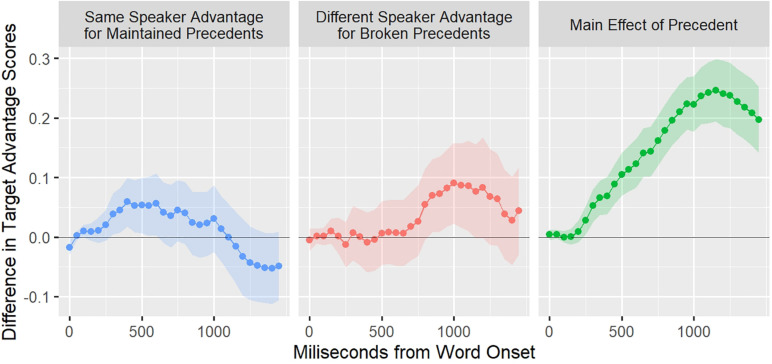
Main results from Kronmüller and Barr’s meta-analysis. Three clear effects are depicted, two of which are speaker-specific and one speaker-independent. Putting together, all of these effects have a different time profile. The blue line represents an advantage for comprehension when the same speaker maintains a precedent. The red line represents the advantage to comprehension when a different speaker uses a new expression. Finally, the green line represents the overall advantage of relying on precedents. This picture is an adaptation to Figure 3 in Kronmüller and Barr (2015).

Figure 1 presents the main results of Kronmüller and Barr’s meta-analysis. The dependent variable was derived from eye-tracking data, specifically the *target advantage score*, which reflects the preference to the target object compared with the other objects across time. *The blue line (left panel) represents a same speaker advantage for maintained precedents*, and it is computed as the difference of the target advantage scores of the same speaker maintained precedent condition and the different speaker maintained precedent condition. Thus, a positive value indicates an advantage for the same speaker when precedents are maintained. As can be seen, there is an early influence of speaker-specific information at approximately 500 ms. However, it decreases as interpretation continues (approximately 1,100 ms). *The red line (middle panel) represents the advantage for broken precedents*. This time, a positive value represents an advantage for the different speaker against the same speaker, in other words, the effect of speaker information when a precedent is broken. For this case, there is a delayed effect compared with the same speaker advantage (starting at 750 ms), but it only increases across time. Finally, *the green line (right panel) shows the advantage of the existence of a precedent*, independently of the speaker. As can be seen, the previous two are relatively small compared with this speaker-independent *effect of precedent*.

The meta-analysis showed clearly that the integration of speaker-specific information has two different timing profiles depending on whether the precedent is maintained or broken. As we will argue below, the time profiles of the effect of speaker-specific information are at odds with both of the accounts presented so far: one-stage and original two-stage.

In effect, the one-stage account sees precedent interpretation as a competition process. In this single process, the different alternatives (in this case referents) “compete” as they gain evidence from various sources of information integrated to comprehension as soon as they are available (Trueswell and Tanenhaus, 1994). As such, it is an instance of the *constraint-based* family of language comprehension models that have been successful in explaining syntactic, semantic, or referential ambiguity resolution (Elman et al., 2004; McRae and Matsuki, 2013). One key prediction from these models is that, if all sources of information point to the same alternative (in this case referents), competition should favor that alternative over all others *at every moment* during the whole process. Indeed, activation of alternatives, which in this particular case is referents, permanently increases in the same direction; if there is a systematic bias toward one alternative, and that bias is constant across the whole competition process, the less activated alternative should not be favored with respect to the other, more activated, alternative.

This pattern should be the case independently of whether or not those informational sources are available simultaneously and also regardless of the strength of association between the alternatives and the informational sources. Therefore, there should be a *monotonic increase* in looks to the target referent when the original speaker maintains a precedent until the competition process ends and one alternative “wins” (meaning that the intended referent is finally selected). The fact that the effect decreases—as can be seen in the green line in Figure 1—cannot be accommodated easily with this prediction.

On the other hand, the mere fact that there is an early effect of speaker-specific information in the maintained condition undermines the central claim of the original two-stage approach, as we have described so far. This approach inherits the view that contextual information, and critically speaker information (including common ground), has a role in an optional and more comprehensive process that monitors for potential misunderstandings (Keysar et al., 2000; Epley et al., 2004; Kronmüller and Barr, 2007). This account has its roots in early views on how parsing a sentence works: first, there is an interpretation based on only syntactic information and only later have a role in the semantic and contextual information (Frazier and Rayner, 1982).

As in the one-stage account, where the early integration of speaker-specific information provides evidence for it, there is also some piece of evidence that favors the original two-stage account. Indeed, even when speaker-specific information has an early influence, what has the most significant impact is whether or not there is an established precedent, independently of the speaker who established it (Kronmüller and Barr, 2015).

### An Extended Two-Stage Account

There is a third possibility, which we defend here, that can reconcile the findings described above: an *extended two-stage account*. Under this idea, the information about the speaker is integrated at two different stages. In the first stage, precedent interpretation could be mediated by information about the conversational context, encoded in episodic memory, and retrieved in a *cue-driven automatic* fashion; such cues include the presence of speakers and salient perceptual features, such as their voice or gender (Horton and Slaten, 2012). Importantly, these episodic memory traces are not the same as common ground and speakers’ beliefs but instead are the basis for inferring common ground (Horton and Gerrig, 2005). The second stage, we propose, might be based on *meta-representations processed by an inferential mechanism*, in line with perspective taking or “mindreading” (for a thorough discussion of mindreading, see Goldman, 2006). As with the original two-stage account, this inferential mechanism, based on information about the speaker, might be triggered as a result of the presence of ambiguity.

To the extent that our reasoning is correct, two moments for the integration of information about the speaker should be identifiable in the time course of the interpretation of precedents. These two moments are apparent in Kronmüller and Barr’s meta-analysis at 900 ms, where the two lines, representing speaker-specific effects, cross each other. The present study tests this possibility, looking directly for two-stage processing of maintained precedents, both relying on speaker-specific information.

### The Present Study

As presented above, the experiments on precedent interpretation so far have shown that referential precedents are interpreted in a one-stage process when they are maintained (Brown-Schmidt; Barr, 2008), but in a two-stage process when they are broken (Kronmüller and Barr, 2007). It might be the case that the second stage of integration of speaker information has not been observed on maintained precedents because the speakers’ perspective is of no use when there is no (or only temporary) ambiguity in the referential situation. With this in mind, we generate a situation of total referential ambiguity if only linguistic information is considered, but that could be disambiguated using information related to the speaker, information about the presence of a linguistic precedent, or both. This design allows us to test predictions from the one-stage model, the original two-stage model, and the extended two-stage processing model for maintained precedents we propose here.

Previous research may have failed to find the integration of speakers’ information in two stages because this information is not necessary to resolve reference. In some cases, there was no ambiguity at all since there was always one referent that was the best candidate for the referring expression. For example, when the instruction was to select “the silver pipe,” there was only one object in the referential domain that best resembled a silver pipe (Metzing and Brennan, 2003; Kronmüller and Barr, 2007). In other cases, ambiguity was momentary at either the level of the noun or the level of the adjective in the noun phrase (Brown-Schmidt, 2009; Horton and Slaten, 2012). For example, the instruction was “select the cat that’s drinking milk,” in a display with two images (e.g., tangrams), one mentioned before as “the cat that’s drinking milk” and the other as the “cat that’s sitting up.” The tasks that show momentary or no ambiguity at all do not require speaker-specific information to be integrated into a second stage of processing, where common ground and speakers’ perspective might be critical to disambiguate. In the present design, in contrast, due to total referential ambiguity that we created, we expect to see information related to the speaker to be used in referential resolution in a second stage as well.

**FIGURE 2:**
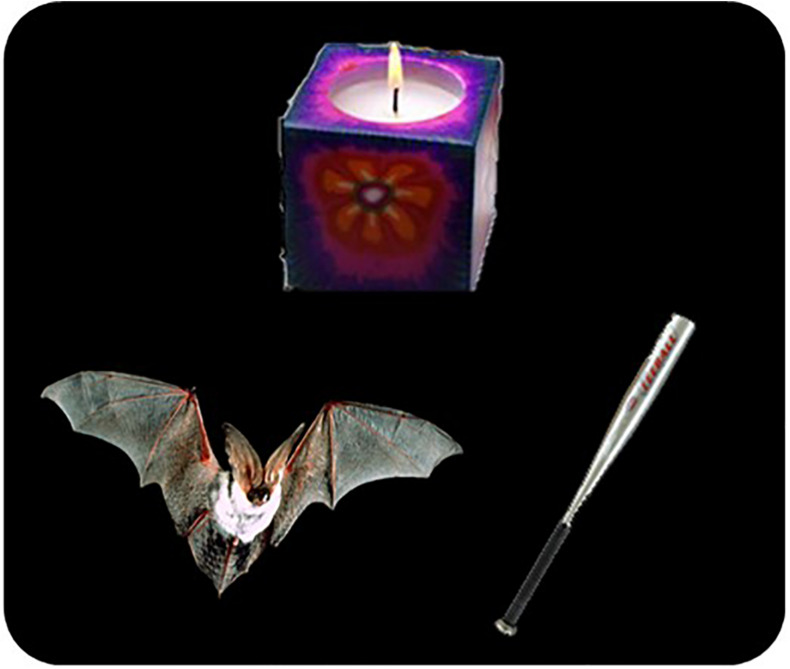
Test trial example. Three objects appear on the screen. Two of them are referential competitors because they can be referred to using the same word: the mammal bat and the baseball bat, which can be both refer to as the bat. The third picture is a distractor, not related with the previous two. The task is to select an object upon hearing an instruction of the type: “click on the bat,” which is ambiguous in this display.

In our experiment, participants saw three pictures of everyday objects on a computer screen and heard an instruction from one of two different speakers referring to one of these objects. In what we called *test trials*, ambiguity was introduced by the presence of two objects that could be named using the same word: a *target* object and a *competitor* object (see Figure 2 for an example of a critical trial). For example, if the instruction was to “click on the bat,” the possible referents were a flying mammal bat (target) and a baseball bat (competitor). Based on which referents were mentioned before the test trials, during what we called *presentation phase*, we generated four experimental conditions by combining two variables, with two levels each, in a within-subjects factorial design. The first variable was *Precedent* with two levels: *Precedent* and *No Precedent.* The second variable was *Speaker*, also with two levels: *Same* or *Different*. In what follows, we briefly describe each condition. A full example for an entire item will be presented in “Materials and Methods” section.

In the presentation phase, before each test trial, the target object (mammal bat) was either referred to or not. The competitor object (baseball bat), on the other hand, was never mentioned in this phase. Also, the target was sometimes mentioned by the same speaker as the one who gave the instructions in the test trials (female) or by a different one (male). Thus, whether or not the target object was mentioned before and by whom gives rise to our four conditions. In the *Same Speaker Precedent Condition* (*SSP*), the target object (mammal bat) was named twice before the test trial by the same speaker (female), establishing a referential precedent. In the *Different Speaker Precedent Condition* (*DSP*), by contrast, the speaker who gave the instruction on the test trials (female) was different from the one who had established the precedent in previous trials (male). In the *Same Speaker No Precedent* (*SSNP*) condition, the same speaker (male) gave the instructions in the presentation phase, but the target referent (mammal bat) was not mentioned. Instead, a different object was mentioned (the candle, the body, or the chest). Finally, in the *Different Speaker No Precedent Condition* (*DSNP*), there was a change of speaker before the test trial, and the target object (mammal bat) was not mentioned in the presentation phase. Specifically, the male speaker gave the instructions in the presentation phase, but the female speaker did it for the test trial. By changing the speaker in the presentation phase (instead of the test trial), we had the same token instruction for all four conditions in each item, allowing strong experimental control. With this design, we tested the hypotheses derived from the competing accounts.

**FIGURE 3:**
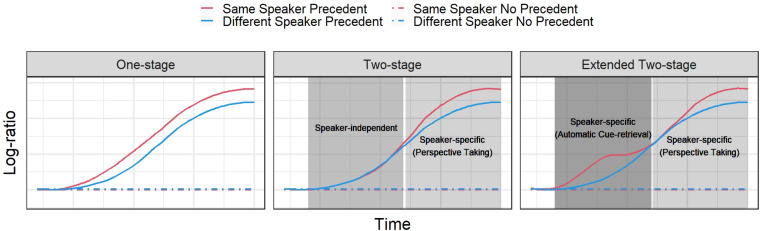
Schematic predictions from each model. Each of the models makes different predictions with respect to the temporal pattern of the log ratio between the target and competitor objects. A value close to zero means no preference to either of the referents. A positive value reflects a preference to the target, and a negative value a preference to the competitor. The left panel shows the pattern predicted by the one-stage model, where there is an early separation between the lines representing the SSP condition and the DSP condition, favoring the former. The middle panel shows the pattern expected for the original two-stage model, where there is an early speaker-independent processing and a late perspective taking process. Finally, the panel on the right shows the predictions from the extended two-stage model, where there is an early speaker-specific processing due to automatic memory processes and a late effect related to perspective taking mechanisms. All models predict no difference in preference between referents across the whole time window when a precedent has not been previously established.

To specify predictions, Figure 3 shows a schematic representation of how eye-tracking data should look like for each account in all conditions. We used a measure that can summarize the competition process between the target and competitor objects. We chose a *log ratio*, which gives a positive value if there was a preference in looks to the target (mammal bat), a negative value if the preference was on the competitor (baseball bat), and zero if there was equal distribution of looks between the objects (we further explain this measure in “Materials and Methods” section). We opted for this dependent variable rather than target proportion of fixations because the latter incorporates all referents in the referential domain in the value, independently of whether or not they are of interest. Log ratios, on the other hand, only consider the referents that are of interest for testing the hypotheses. In our case, these are both referential competitors.

First, all competing accounts predict an advantage to the target object in both precedent conditions, independently of the speaker. In Figure 3, this effect is represented in the difference between the two green lines and the two red lines. The first two go up, whereas the second two remain at zero all the time (black thin line). Indeed, without a precedent associated with the objects, there should not be any preference for one of them, making the log ratio close to zero. Where the accounts should contrast is in the difference between the two speaker conditions when there is a precedent, namely, the SSP and DSP conditions. The one-stage account predicts an early separation of the curves since two cues are pointing to the target in the SSP condition, a linguistic cue in the form of a referential expression, and a contextual cue in the form of speaker’s identity. In the DSP condition, in contrast, only the linguistic cue points to the target. Importantly, and as we argue above, both curves should increase monotonically.

The original two-stage account predicts a late separation of both precedent curves, since in the first stage only the linguistic information is processed. The contextual information is considered in the second stage through a perspective-taking process.

The pattern that would be consistent with the extended two-stage account should present two clear moments at which speaker information contributes to the preference to the target object over and beyond the existence of a precedent. The rightmost panel in Figure 3 shows such a pattern. The early separation of SSP and DSP curves would reflect the automatic cue-driven process, whereas the late separation would be the consequence of an inferential process based on perspective taking, which would be triggered by the presence of ambiguity.

## Materials and Methods

### Participants

Fifty-six undergraduate students (age range: 18–24 years) participated in this study in exchange for course credit for an introductory course in psychology at the University of California, Riverside. All of them had normal or corrected-to-normal vision and were native English speakers. Thirty-nine of the participants were female.

### Design

The experiment had a 2 (Same Speaker or Different Speaker) × 2 (Precedent or No Precedent) within-subjects factorial design.

### Materials

**FIGURE 4:**
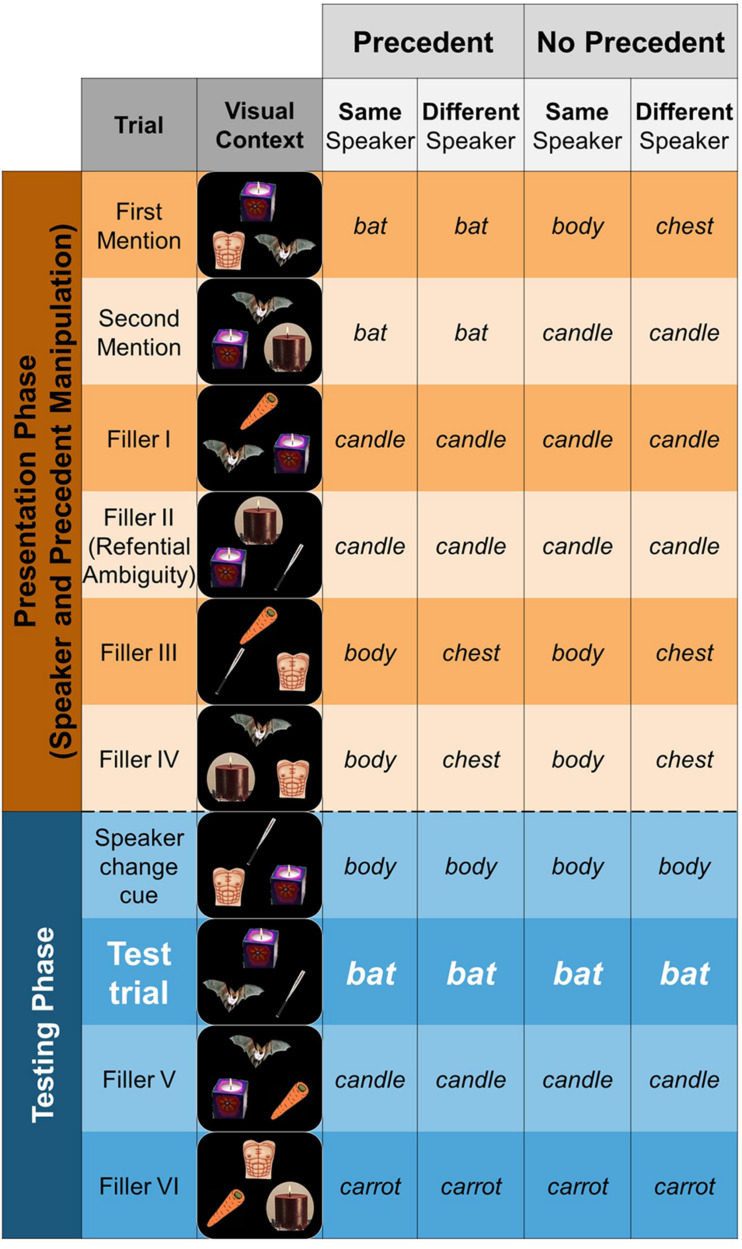
A schematic representation of a 10-trial sequence in which a test trial is embedded (either in the eighth or ninth position). The sequence is divided into two phases: Presentation (first six trials in orange) and the Testing (subsequent four trials in blue). Precedent, or its absence, is established during Presentation, producing the Precedent vs. No Precedent conditions. During Testing, speaker is either maintained or changed (relative to Presentation), resulting in the Same vs. Different Speaker condition.

**FIGURE A1:**
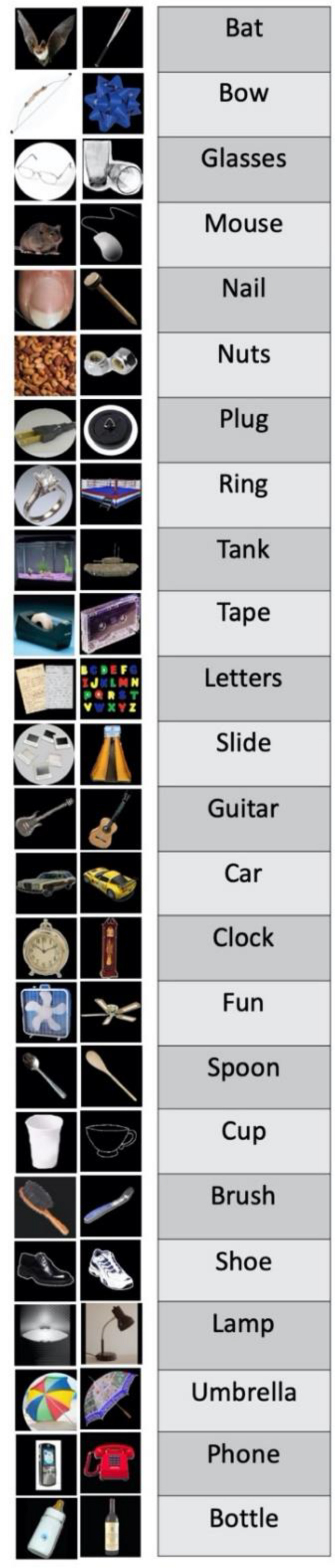
List of referential competitors materials.

We created 24 items. Figure 4 presents an example of an item and its corresponding elements (pictures and sound files): each item consisting of six pictures of everyday objects and 16 sound files with instructions (e.g., “Click on the bat”) recorded by two different speakers. Two of the objects were referential competitors, i.e., objects that could be referred to using the same English word. For the sake of exposition, we called one these objects “target” and the other “competitor.” For example, as in Figure 2, a mammal bat and a baseball bat can both be referred to as “the bat” (see Figure A1 for the complete list of referential competitors). One of the remaining four pictures was used to introduce salience to the speaker manipulation in a “Speaker Change Cue” trial (see “Procedure” section and Figure 4). The three remaining pictures were fillers. Each item was a sequence of 10 displays, presenting three pictures at the same time. Therefore, we use different combinations of the six objects pertaining to an item to generate the 10 displays of each item. For the sake of exposition, we will refer to each display as a “trial.”

On each trial, participants heard sound files, containing the instructions, depending on the experimental condition and the type of trial. Using pre-recorded speech—instead of live instructions from a speaker—allowed us to present the same instruction token in every condition. With this feature, we avoid possible confounding variables, such as paralinguistic cues (a point we come back to in the discussion). We used female and male voices for the speaker manipulation, as these have been shown to boost talker-specific effects in lexical recognition tasks (Creel et al., 2008). For half of the items, the female voice gave the instruction in the critical trial and the male voice for the other half.

### Procedure

Participants were tested individually in a quiet and semi-dark room. They sat facing a projection of a computer screen on the wall. In between the projected screen and the participants, there was a table with an ISCAN ETL-400 remote eye tracker (60 Hz sampling rate). Participants were informed that they would play a communication game in which they would have to follow the directions of two different speakers. Each time, one of the speakers would instruct them to select one picture among the three images presented on the screen. They were told that each speaker recorded the instructions in a different session so that they might refer to the objects differently between them. We also told them that because speakers did not interact with one another, they were uninformed of each other’s precedents. Additionally, participants were led to believe that the order of the instructions they heard was the same as the order that they were recorded.

**FIGURE A2:**
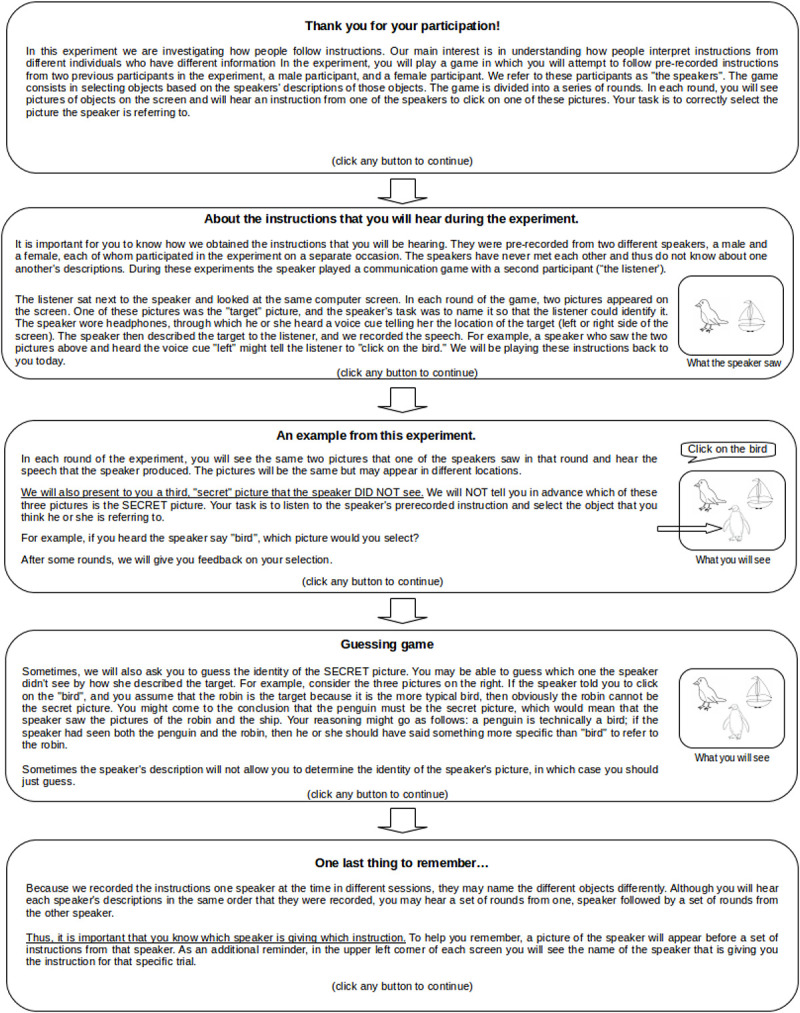
Schematic of the instructions given to participants before the experiment started.

Participants were also led to believe that when the speakers recorded the instructions, they could only see two of the three pictures on the screen. This feature was introduced for two reasons. First, we wanted to make the situation more natural and cooperative from the point of view of the listener. Since the instructions were ambiguous between two objects, a speaker normally would disambiguate their utterances by further specifying the intended referent. For example, in the presence of a mammal bat and a baseball bat, a speaker would say “the mammal bat” and not merely “the bat” if she needed to specify a referent uniquely for an addressee. The second reason concerned the implementation of a guessing game, where the participant had to guess which picture on the screen the speaker did not see. This guessing game provided data about the possible explanations that participants generated to explain why the speaker was ambiguous; specifically, if they selected one of the competitors (mammal bat or baseball bat), then it would be clear that they believed the reason was that when producing the utterance the speaker could not see the other object that was the source of the ambiguity. Figure A2 depicts a schematic representation of the instructions given to the participants (the real instructions were given in different sequential displays on the screen).

To make the experimental situation more credible, right before the trials started, participants engaged in a role play where they gave instructions on which object to select to the experimenter. With this process, we led them to believe that they were experiencing a similar situation as the one that the speakers they were about to hear experience when recorded the instructions. The only difference was that the addressee was the experimenter instead of a real participant. After the instructions were recorded, they were played back to the participant to practice the actual task.

### A Description of an Item: Phases and Type of Trials

Each test trial was embedded in a 10-trial sequence, corresponding to one item. There were three other types of trials in this sequence: the *presentation trials*, the *filler trials*, and a *speaker-change cue trial* (see Figure 4 for a schematic representation). The sequence was divided into two phases, a *Presentation Phase*, consisting of six trials, and the *Testing Phase*, with four trials. As we described above, the precedent manipulation was implemented during the Presentation Phase via the presentation trials, where the target referent (e.g., the mammal bat) was referred to either twice (precedent conditions) or never (no precedent conditions). In both presentation trials for the precedent conditions, the speaker referred to the mammal bat as “the bat.” In the presentation trials for the no precedent conditions, the speaker referred to one of the filler objects in the display (e.g., the chest and the candle in Figure 4). The speaker manipulation was implemented using the transition between the presentation and testing phases. In the same speaker conditions, the speaker that provided instruction in the presentation phase (e.g., female) continued giving the instructions in the testing phase. In the different speaker conditions, by contrast, the other speaker (e.g., male) provided the instructions for the presentations phase. As we said above, this feature allowed us to have the same token instructions in each item.

Speaker-change cue trials were included to add salience to the (same vs. different) speaker manipulation. On these trials, one object was referred to using a different name as in the presentation phase when there was a speaker shift. For example, if an object was called “the chest” by one speaker during the filler trials in the presentation phase, then the same object was called “the body” by the other speaker in the testing phase (see Figure 4). By doing this, we hoped participants would notice the shift and strengthen their belief that the speakers were unaware of one another’s referential commitments. Additionally, we added a picture of the speaker that was about to give instructions. Finally, to further mask the critical trial, we added a filler trial with two referential competitors.

The test trials took place in the testing phase. As we have described, ambiguity for these trials was introduced by the presence of two objects that could be referred to using the same expression: a mammal bat (target) and a baseball bat (competitor). Therefore, the instructions for the critical trials contained the same linguistic item that can be applied to either competitor, e.g., the word “bat” in the instruction “click on the bat.” The task of the participants was to select one of the three objects using a computer gamepad. Participants’ eye movements and picture selections were recorded. Given our goal in this study, the data we were interested came only from the test trials.

Finally, after the participants made their selection, the guessing game took place. In the guessing game, the three pictures appeared again on the screen, and the task of the participant was to select the one that they believed the speaker could not see when the speaker gave the instruction. In order to cover the identity of the test trials, the guessing game was also implemented after two other trials in each item during the presentation phase.

### Data Analysis

Selection data (the object the listeners chose) and reaction times to that decision were analyzed using a mixed-effects regression with cross-random effects (Baayen et al., 2008), with subjects and items as random effects and Speaker and Precedent as fixed effects. We tested the effects using a model comparison approach, where a full model is compared with a restricted model without the fixed effect of interest and a chi-squared statistic is reported. The full model contained all random intercepts and slopes. Random correlations were excluded because the model would not converge (Barr et al., 2013). For the selection data, we performed a logistic regression because the total of selections of the unrelated object was 3 out of 1,344 trials, making it, in practice, a dichotomous variable. For reaction times, we log-transformed the data to ensure normality in its distribution to conduct the statistical test. We, however, report the raw data, i.e., in milliseconds.

Selection and reaction time inform us about the result of the comprehension process. However, they do not inform us about the temporal profile of the process of interpreting a referential expression. Since our hypotheses concern a specific temporal pattern determined by the underlying cognitive processes, we conducted a growth curve analysis (GCA). This analysis can capture a temporal profile by fitting a polynomial function and can provide estimates for each component of it (Mirman et al., 2008; Mirman, 2014). For this particular data, we fitted a third-degree polynomial function, capturing the linear, quadratic, and cubic components.

To compute the *p*-values for each of the terms in the polynomial function, we decided to conduct a resampling test (Carsey and Harden, 2013; see Kronmüller et al., 2017; Kronmüller and Noveck, 2019, for the use of resampling tests to conduct inferential statistics on eye-tracking data). As we mentioned above, since our main interest was the pattern of transitions between the target and competitor objects, our dependent variable was the log ratio of the looks to the competitor over the target (see Arai et al., 2007). In this measure, positive values reflect more looks to the target than to the competitor object, negative values reflect a relation in the opposite direction, and values close to zero show no preference to any of them. We computed this log ratio by fitting baseline-category multinomial logistic regression (Agresti, 2003).

The algorithm to compute the *p*-values had several steps. In the first step, we obtained the regression coefficient for each of the polynomial terms for the original dataset (Mirman, 2014). Second, we draw a Monte Carlo random sample of 9,999 permuted datasets (Carsey and Harden, 2013; Berry et al., 2016). Third, we created a null hypothesis distribution of the regression coefficients for each polynomial term, to each permuted dataset, and we stored them. Therefore, we obtained 9,999 coefficients for each polynomial term. And fourth, we compared the original regression coefficients with the null hypothesis distribution by calculating the proportion of coefficients that were larger or smaller. These proportions are the *p*-values. Since we had specific hypotheses about (a) the cubic pattern between the Same Speaker Precedent and Different Speaker Precedent conditions and (b) the linear pattern of the effect of precedent independently of the speaker, we performed one-tailed tests. We computed two *p*-values for each term, one for subjects (*p*1) and one for items (*p*2).

## Results

Participants followed the instructions for the experiment and were attentive to the speaker manipulation and the task. Evidence for this can be found in the relation between the object that participants selected in the main task and the guessing game. When they chose the target (e.g., mammal bat) in the main task, they selected the competitor (e.g., baseball bat) in the guessing game 90.0% of the time. And when they selected the competitor in the main task (e.g., baseball bat), they chose the target (e.g., mammal bat) in the guessing game 78.3% of the time. This pattern also shows that, even though listeners heard pre-recorded expressions, they expect the speakers to disambiguate if necessary; in other words, listeners’ pragmatic expectations were not compromised during the experiment.

### Target Selection Data

Participants preferred the picture named before as the target: it was selected 78.0% of the time in comparison with 54.3% of the time when there was no precedent. This main effect of Precedent is statistically significant [χ^2^(1) = 30.341, *p* < 0.0001]. Also, there is a reliable interaction [χ^2^(1) = 7.342, *p* = 0.007] driven by a higher selection of the target object when the same speaker established the precedent (82.1%) than when a different speaker established it (72.8%) [χ^2^(1) = 6.904, *p* = 0.009]. No statistically reliable simple effect was found between the two speaker conditions when there was no referential precedent [χ^2^(1) = 1.737, *p* = 0.188]. Table 1 presents the percentage of object selection by experimental condition.

**TABLE 1:** Percentage of selection of each object across conditions.

	Same speaker		Different speaker	
		
	Precedent	No precedent	Precedent	No precedent
Target	82%	52%	74%	57%
Competitor	18%	48%	26%	43%
Unrelated	0%	0.03%	0.1%	0%

### Reaction Times

Table 2 presents the mean reaction times by conditions. Participants were faster to make their selection when there was a precedent (*M* = 1,530 ms; *Mdn* = 1,212 ms) than when there was no precedent (*M* = 1,720 ms; *Mdn* = 1,375 ms). This difference is statistically reliable [χ^2^(1) = 6.375, *p* = 0.012]. In contrast, there was no main effect of speaker: the mean reaction times for the same speaker (*M* = 1,587 ms; *Mdn* = 1,267 ms) were not statistically different [(χ^2^(1) = 2.037, *p* = 0.154] from the mean for the different speakers conditions (*M* = 1,663 ms; *Mdn* = 1,298 ms). Also, the interaction was not statistically significant [χ^2^(1) = 0.842, *p* = 0.359]. The mean and median for each condition were as follows: Same Speaker Precedent (*M* = 1,536 ms; *Mdn* = 1,230 ms); Different Speaker Precedent (*M* = 1,525 ms; *Mdn* = 1,209 ms); Same Speaker No Precedent (*M* = 1,638 ms; *Mdn* = 1,328 ms); and Different Speaker No Precedent (*M* = 1,802 ms; *Mdn* = 1,387 ms).

**TABLE 2:** Reaction times of object selection across conditions.

		Same speaker	Different speaker
	Main effects	1,587 ms	1,663 ms
Precedent	1,530 ms	1,536 ms	1,525 ms
No precedent	1,720 ms	1,638 ms	1,802 ms

In summary, the selection data show that by the end of the interpretation process, listeners’ interpretation of referential precedents has a speaker-specific component. This finding is consistent with the one-stage, original two-stage, and extended two-stage accounts. The reaction time data portray a slightly different picture. Participants’ final selection took longer in the No Precedent conditions than in the Precedent conditions, independently of the speaker. Then, even when more competition would be expected in the DSP condition than in the SSP condition (since more informational cues point in the target direction in the latter than in the former), participants do not take longer to make their selection.

Notwithstanding, we can truly differentiate between the three accounts by looking at the interpretation process as it unfolds and observe the influence of the speaker information as it emerges over time. We now present the eye-tracking data.

### Eye-Movement Data

**FIGURE 5:**
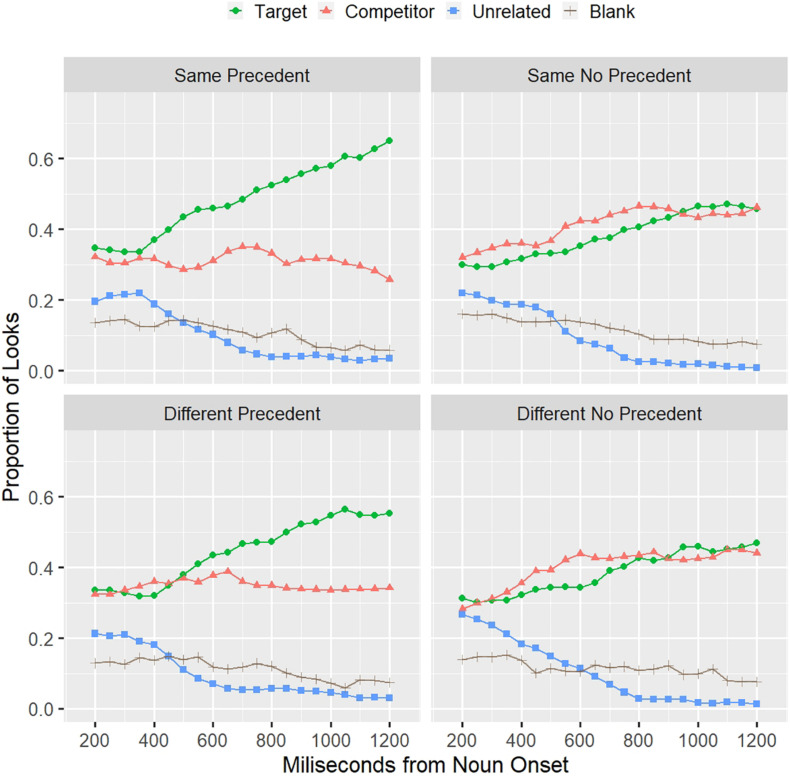
Proportion of looks to each object and the blank part of the screen across the four conditions. Data are time-locked at the onset of the noun in the referring expression (e.g., “the bat”).

Figure 5 shows the proportion of looks to each object from the onset of the critical noun in the test trial (e.g., “bat”) to 1,300 ms after it. Fifty percent of trials terminated within this time window because subjects had made their selection. The graph starts at 200 ms from the onset of the critical word since it is the time that has been estimated to program an eye movement (Matin et al., 1993). Therefore, from 200 ms after the onset, eye movements can be driven by the linguistic input, in this case, the noun in the noun phrase (e.g., “bat”). The mean duration of nouns in the stimuli set was 455 ms (range 351–806 ms).

From the graphs for each condition, it can be observed that the divergence between the target and the competitor objects starts earlier in the SSP condition than in the DSP condition. It is also clear that when there is no precedent, participants cannot overcome ambiguity, looking equally to both—the target and the competitor—throughout the time window.

**FIGURE 6:**
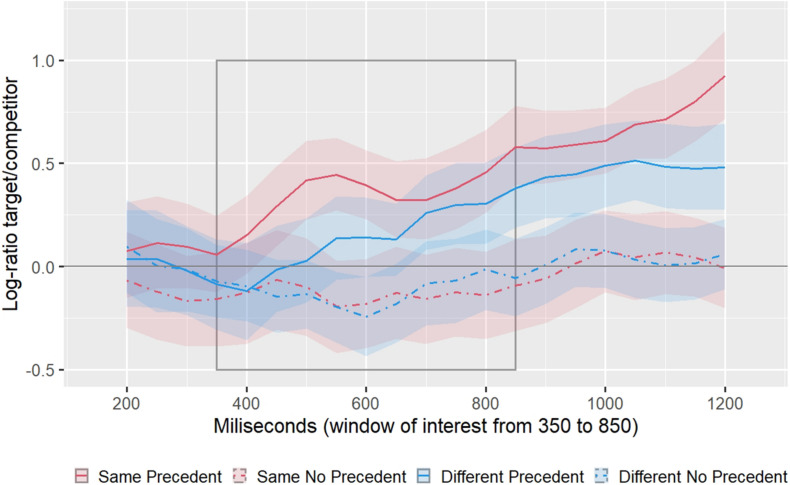
Log ratio of fixations to the target over the competitor between 350 and 850 ms after the onset of the noun in the referring expression (e.g., “the bat”). The solid line represents the mean, and the shaded area represents 95% confidence interval, both calculated by bootstrapping subjects. The gray rectangle represents the window of interest.

To test our two hypotheses, we conducted our statistical analyses on the log ratios in a specific window. Figure 6 shows these log ratios in the time window of interest, which we define between 350 and 850 after noun onset. We determined this window following two criteria. The first one was to match the timing on which the same-speaker benefit appears in Kronmüller and Barr’s (2015) meta-analysis (see Figure 1). The second criterion was to ensure a clean cubic pattern for the analyses so that the first time point was not be preceded by a time point with a larger log ratio and the end time point was not be followed by a lower log ratio.

One hypothesis was the specific non-monotonic pattern for the SSP condition in contrast to the DSP condition, which would reflect the influence of speaker specificity in two different stages. To test this hypothesis, we compared the SSP and DSP conditions. As can be observed, the SSP condition presents a cubic pattern, which reflects the influence of speaker information at two stages. This pattern is not observed in the DSP. The cubic component of the polynomial is observed in the SSP condition and not in the DSP condition (*p*1 = 0.027, *p*2 = 0.031). Neither the linear (*p*1 = 0.703, *p*2 = 0.670) nor the quadratic term was significant (*p*1 = 0.689, *p*2 = 0.652).

The other hypothesis was related to the effect of precedent, independently of the speaker. As can be observed in the graphs, there is an increase in log ratios (more looks to the target than to the competitor) in both precedent conditions than in the no precedent conditions. The linear component of the polynomial for the main effect of precedent is significant by subject (*p*1 = 0.013) and marginally significant by item (*p*2 = 0.085). Neither the quadratic nor the cubic was significant.

The only other effect that reached statistical significance was a main effect of speaker in the cubic term (*p*1 = 0.031; *p*2 = 0.032), favoring the Same speaker conditions, which we had not hypothesized.

## Discussion

Interlocutors frequently rely on referential precedents to reach mutual understanding regarding the entities being referred to across a conversation. These referential precedents carry information that goes beyond the conventional meaning of the words, as they are encoded along with information related to the history of a conversation with specific partners. In this study, we contrasted predictions from a one-stage model of referential precedents, an original two-stage model, and an extended two-stage model, and we focused on the integration of speaker-specific information. The first model, as a version of constraint-based models of language processing, predicts—in the context of our design—a monotonic increase for preferences to the target when speakers’ information is added to lexical and precedent information. The original two-stage model predicts only a late integration of speaker information. The model we defend here, the extended two-stage approach, predicts that information about the speaker has its influence at two different moments. Thus, our rationale was to distinguish these accounts based on a temporal dissociation of the effect of speaker information during the interpretation of precedents. We generate an experimental situation of referential ambiguity that could be resolved using speaker information, the presence of a precedent, or both. Consistent with the extended two-stage account, we found that speaker information has an influence at two different moments during online interpretation: descriptively speaking, we can see that at ∼350 ms after noun onset, an SSP advantage begins, only to decrease at ∼550 ms and reappear again at ∼700 ms up to the end of the time. This drifting reflects the cubic pattern predicted by the extended two-stage account for the SSP condition.

We propose that the first moment where speaker information is observed might be the result of an automatic cue-driven retrieval process of episodic memory traces that encode contextual information (among which are previous encounters with the precedent and the speaker). The second moment when the speakers’ information “kicks in” can be seen as the result of mentalizing or perspective-taking mechanisms for inferring communicative intentions based on speaker’s beliefs and knowledge. One result that might have been expected is a difference in reaction times between the two speaker conditions. In effect, there is evidence that speaker voice have an effect on lexical access (Goldinger, 1998). We are not clear why this effect is not observed in our data. One possible explanation is the strength of the precedent effect that might override the effect of speaker. Another possible explanation is that reaction times are rather large in our experiment compared with Goldinger’s, mainly because of task differences and the existence of ambiguity in our paradigm. These large reaction times could have hidden the effect of speaker.

### How Can These Results Be Interpreted in Terms of Mechanisms?

In the context of this experimental design, the non-monotonic pattern of the influence of speaker is inconsistent with a one-stage process that would reflect the functioning of a constraint-based mechanism (Brown-Schmidt, 2009; Brennan and Hanna, 2009; Brown-Schmidt and Hanna, 2011). Basically, the two sources of information that could be used to disambiguate the referential expression “the bat”—the presence of a precedent and speaker identity—pointed to the same alternative, in our example, the mammal bat. From a one-stage model, the activation reflected in eye movements toward the target object should increase systematically until the final selection, but this pattern was not observed in our data. Instead, we found an early but transient effect of speaker that disappears and appears again progressively and was maintained until final referential commitments. Similarly, a constraint-based mechanism would predict a fast resolution of the ambiguity in cases were more cues point in the direction of a referential interpretation, which was the case of the Same Speaker Precedent condition compared with the Different Speaker Precedent condition (in the former, the speaker cue was present, and in the latter, it was not present). However, there was no difference in reaction times for final selection between these two conditions, showing further inconsistent evidence for this model.

It is relevant to stress the importance of the prediction of monotonicity for the design presented here, since constraint-based models have been subject of criticisms for being unfalsifiable. This critique has been considered somehow unfair by the proponents of these models (Elman et al., 2004). The criticism points that these models could, in theory, accommodate any pattern of results, since they have many free parameters, such as the weights between constraints and the alternative interpretations, the activation of those constraints, and the timing of their availability. To address this critique in the context of sentence comprehension research, the main effort has been to explicitly set those parameters by consulting corpora and conducting behavioral experiments (McRae et al., 1998; Spivey and Tanenhaus, 1998). Unfortunately, such parameterization has not been pursued in the research on dialogue and speaker specificity in reference resolution.

It has been acknowledged that such an enterprise is hard to fulfill in this context, and thus manipulations of speaker salience—mainly through changing the experimental task for making it more interactive—have been the preferred practice to test the model (Brown-Schmidt and Hanna, 2011). We argue that our design can test direct predictions without specifying weights, activation, and availability and without relying on making speaker information more salient. We achieve this by creating a totally ambiguous situation if contextual information is not considered, and in which all constraints point in the same direction. It is important to clarify that we are not saying that these models cannot show non-monotonic patterns but that in our design they should not.

More generally, there is a conceptual issue that makes a constraint-based approach to dialogue not totally adequate and that is related with the inferential nature of communication (Grice, 1957; Sperber and Wilson, 1987; Clark, 1996). It is not clear how these models could deal with meta-representations, such as speakers’ beliefs about mutual knowledge and common ground. Common ground and speakers’ beliefs are not just simple representations but also representations of mental states or meta-representations, and it is not clear how they can be encoded in episodic memory (Horton and Gerrig, 2005). An interesting proposal has been put forward recently to account for the role of meta-representations on reference production and resolution, where a meta-representation, once inferred, could be stored as any other representation, and from there, it can influence language processing as any other representation (Horton and Brennan, 2016). The representational format of common ground in memory is an interesting area of research that is critical to understand perspective taking in conversation (for discussion on the role of memory in common ground during conversation, see Brown-Schmidt and Duff, 2016).

How could the present results be accommodated under an extended two-stage account? We take the view that precedent interpretation involves two different mechanisms, each one related to one of the two stages. First, interpretation of precedents can be characterized as mediated by contextual information encoded in episodic memory. This information includes the history of the precedent (time, place, and identity of the speaker, including voice and gender) since it was first established (Metzing and Brennan, 2003). In short, it is some sort of source memory (Ryskin et al., 2015). The mechanisms for the integration of this information can be characterized as an automatic cue-driven retrieval process (Horton and Slaten, 2012; Horton and Gerrig, 2016) or episodic priming (Barr et al., 2014).

The second stage could reflect the functioning of an inferential mechanism that takes into account speakers’ beliefs to explain their referential behavior. In the case of the present study, the behavior corresponds to the use of a certain linguistic form to bring into joint attention a particular entity in the context of ambiguity—where the linguistic form is insufficient to uniquely identify a referent as is required by the use of definite reference. The specific characteristics of this mechanism are still unclear. In some sense, it looks like a full mentalizing process cannot really account for the data, specifically for the big effect that the presence of a precedent has on comprehension, independently of who established it. Indeed, if participants had taken speakers’ perspective in full, the Different Speaker Precedent condition should look similar as the other two No Precedent conditions; a new speaker could plausibly refer to either the mammal or the baseball bat by uttering “the bat.” One possible line of explanation can be found in the philosophical discussion on the mechanisms for predicting and explaining others’ behavior or *mentalizing*. In particular, *simulation theory* (Gallese and Goldman, 1998; Goldman, 2006) postulates a mechanism where people attributing mental states to others use their own mental states instead of meta-representations of the others’ metal states. Translated to language interpretation, a listener could use her own mental states as proxies to the speaker’s and from there could make inferences to explain the referential behavior. Using their own mental states as proxies can bias interpretation in an egocentric way (Goldman, 2006). Moreover, this simulation can be characterized as driven by heuristic reasoning (Gigerenzer et al., 1999; Todd and Brighton, 2016). This could be considered a shortcut to infer communicative intent that relies on less information but still generates *good enough* interpretations (Ferreira and Patson, 2007), which are efficient despite the time and cognitive demands of a conversation. In any case, whether full mindreading or simulation, an inferential mechanism ought to be included in an account of referential communication in dialogue. Indeed, one of the major conceptual advances in the study of communication in psychology and human sciences is to consider it an inferential process in nature (Grice, 1957; Sperber and Wilson, 1987).

### Limitations and Future Research

A potential limitation of the present study is related to the “social” situation in the experiment. In order to gain experimental control, we decided to use pre-recorded expressions instead of the presence of two “live” speakers. As we mentioned before, this allowed us to measure responses to exactly the same token instruction, avoiding undesirable noise to the data. This is in line with Kuhlen and Brennan’s (2013) suggestions on the adequate use of confederates in dialogue research. Concretely, they argue that “controlled” dialogue is best exploited when the confederate speaker is meant to initiate the interaction and when testing hypotheses related to an unusual situation (i.e., referential ambiguity in the present study). However, this situation is not as naturalistic as desired when studying dialogue, and this has been an important argument when testing predictions from a constraint-based mechanism. Indeed, the salience of speaker information might be weaker compared with the presence of a live speaker in the context of an interactive task (Brown-Schmidt, 2009; Brown-Schmidt, 2012; Brown-Schmidt et al., 2015). However, in the light of a recent meta-analysis, this claim should be qualified, since the argument only holds with a selective inspection of the literature, i.e., focusing on certain experiments and only some conditions (Kronmüller and Barr, 2015). Moreover, in the present experiment, we emphasized the saliency of the speaker through the inclusion of broken-precedent trials, by having speakers of different gender and by showing the speakers’ pictures.

Indeed, early effects of speaker for maintained precedents have been found in interactive (Brown-Schmidt, 2009) and non-interactive settings (Kronmüller and Barr, 2007; Horton and Slaten, 2012). But they have not been found in interactive ones as well (Metzing and Brennan, 2003). The fact that we did find speaker effects is a compelling argument for the reliability of our results. Indeed, even without a real interactive situation, listeners can make speaker-specific interpretations. Moreover, higher (or lower) salience as a result of the presence of a live speaker and interactivity of the situation cannot explain the integration of speaker information in two different moments, as we show here. We do agree, however, that a more ecological situation is important to test hypotheses related to cognitive mechanisms in dialogue, but we also believe that experimental control is necessary to test hypotheses that differentiate effects at the scale of milliseconds.

The present study can motivate future research to further investigate the nature of the cognitive mechanisms involved in precedent interpretation. Given that our proposal sought to conciliate previous findings in the literature, it is necessary to generate new experimental situations to further understand the nature of the mechanisms underlying the two stages. One possibility is to generate a condition of cognitive load. Since we have characterized the integration of speaker information in the first stage as an automatic retrieval process, and in the second as an inferential one, the former should be less affected than the latter by higher memory demands. Evidence in line with this differential effect of cognitive load has been found before, but not in the context of full ambiguity (Kronmüller and Barr, 2007).

Also, there is a need to move forward and generate computational models of the mechanisms being tested. As mentioned before, modeling has been of major importance for testing predictions of models in sentence comprehension (McRae et al., 1998; Spivey and Tanenhaus, 1998). There are recent efforts to mathematically model and test predictions in the context of pragmatic inference and referential communication in dialogue (Frank and Goodman, 2012; Heller et al., 2016). However, they stress the normative character of pragmatic inference based on Bayes’ rule. We believe that these efforts are important but that a more mechanistic approximation to computational modeling is needed, for example, with ACT-R architecture (Anderson et al., 2004; Hendriks, 2016).

## Conclusion

In conclusion, referential precedents appear to be carrying a great amount of information related to lexical, indexical, and contextual information, all of which helps in reducing ambiguity and facilitates mutual comprehension. The present research suggests that such a rich array of information distinctions calls for more than one cognitive mechanism, some of them related to memory processes, while others related to social cognition.

## Data Availability Statement

The raw data supporting the conclusions of this article will be made available by the authors, without undue reservation, to any qualified researcher.

## Ethics Statement

The studies involving human participants were reviewed and approved by the University of California, Riverside. The patients/participants provided their written informed consent to participate in this study.

## Author Contributions

EK designed the study. EK and EG analyzed the results and wrote the manuscript. Both authors contributed to the article and approved the submitted version.

## Conflict of Interest

The authors declare that the research was conducted in the absence of any commercial or financial relationships that could be construed as a potential conflict of interest. The reviewer HK declared a past co-authorship with one of the authors EG.
